# Supplementary material for: Are carbon emissions trading and green financial instruments synergistic? -Comprehensive quantitative research based on content analysis
Source: PLoS One. 2024 Mar 7;19(3):e0298601. doi: 10.1371/journal.pone.0298601 (PMC10919601; doi:10.1371/journal.pone.0298601)
Supplement: S1 Data — (ZIP) [file pone.0298601.s001.zip › Data/地级市Z_F工作报告.zip!/╜¡╬≈/╛░╡┬╒≥╩╨/└┤╫╘╣1⁄2╓┌║┼í╛┬φ┐╦╩2╛▌═°í┐_2015.docx]

各位代表：

　　现在，我代表市人民政府向大会作工作报告，请予审议，并请各位政协委员和列席会议的同志提出意见。

　　一、2014年工作回顾

　　过去的一年，面对经济下行压力，在省委、省政府和市委的坚强领导下，全市上下紧紧围绕“千年古镇、世界瓷都、生态之城”的战略定位，全面实施工业、招商引资、城市建设“三大战役”，迎难而上，务实创新，实现了经济社会平稳较快发展。经过一年的努力，“三大战役”初战告捷，随着战役的深入推进，成效将充分显现。全年生产总值738.21亿元，增长8.8%；财政总收入101.5亿元，增长9.6%，其中公共财政预算收入82.1亿元，增长11.4%；固定资产投资622.54 亿元，增长15.6%；外贸出口7.58亿美元，其中生产型企业出口7亿美元，增长14.6%；社会消费品零售总额239.88亿元，增长12.5%；城镇居民人均可支配收入26625元，增长9.7%；农村居民人均可支配收入11547元，增长11.4%；单位生产总值能耗下降3.6%；人口自然增长率低于7.29‰。工作及成效主要体现在八个方面：

　　（一）工业发展势头良好。以“一线五点”工业走廊为主平台，全面打响工业强攻战，全部规模以上工业增加值273亿元，增长11.3%。工业产业加速集聚，直升机及通航、汽车及零部件等六大优势产业占比76.34%，提高7.24个百分点。航空产业实现总产值144.43亿元，增长7.62%。高新区由15平方公里扩展到20平方公里，包括3家整机厂在内的15家航空企业落户园区，其中4家投产、5家开工。昌飞总装园启动厂房主体工程建设。北汽集团与江直投达成战略合作协议，厂房主体工程土地平整到位，并租赁厂房生产6架整机。汽车产业实现总产值101.03亿元，增长26.9%。北汽集团顺利重组昌汽，新基地厂房和生产线建设全面启动，10家零部件供应商签订入驻协议；昌汽老厂区通过技改导入新车型并实现当年投产。陶瓷产业总产值291.6亿元，增长17%。陶瓷工业园区由3.73平方公里扩展到8.1平方公里，名坊园一期基本建成，20余户手工制瓷企业落户，红叶陶瓷、邑山陶瓷等企业加快入园，盛景、景浮宫等19个亿元陶瓷项目投产。此外，焦化集团实现营业收入107亿元、出口3亿美元；华意新600、世龙实业氯化亚砜扩产、大瓷庄骨质瓷等26个重点项目竣工投产；恒盛化工年产27万吨表面活性剂和洗涤用品等亿元项目开工。

　　（二）“三农”基础不断夯实。农业总产值82.67亿元，增长4.5%。粮食总产62.9万吨， 实现“十一连增”。蔬菜和茶叶产值合计完成20.29亿元，增长5.78%。市级以上龙头企业发展到109家，规模以上休闲农业企业140余家。39个乡镇完成乡镇总规和控规修编，7个中心镇、3条美丽乡村综合示范带、233个新农村点建设扎实推进。投入8.9亿元实施农田水利基本建设，除险加固病险水库113座，堤防加固、河道疏浚和渠道维修1600余公里，新增、恢复农田灌溉8.55万亩，建设高标准农田4.3万亩。新增造林4.68万亩，超额完成省下达计划。

　　（三）开放合作不断扩大。积极策应“一带一路”和长江经济带等国家战略，全力打好招商引资大会战并取得丰硕成果。承办江西（景德镇）直升机产业集聚发展推介会，积极参加低碳会、赣港会、赣台会、泛珠会等重大活动，并赴温州、台州、泉州等多地开展招商。利用省外5000万元以上项目105个，实际进资320.18亿元，增长16.3%；利用外资1.55亿美元，增长10.5%。组织100多家企业参加广交会、华交会，在俄罗斯、意大利、巴西等国开展对外交流、拓展国际市场。获得商务部支持并签订了对外经贸发展合作框架协议。与上饶、鹰潭达成赣东北扩大开放合作框架协议。成功举办2014瓷博会，质量和效益均比上届有所提高，并以瓷博会为平台，举办了“海上丝绸之路”主题展、“瓷之源、茶之乡”海上丝绸之路学术论坛等主题活动。

　　（四）重大项目成效显著。九景衢铁路建设全面启动，征地基本完成，房屋征收有序推进，北站房及站前广场即将开工。浯溪口水利枢纽工程提前截流，大坝主体工程形象进度过半。城防工程瓷都大桥至观音阁段一期完成1.4公里堤防建设，吕蒙堤完成工程进度22.1%。赣东北综合物流园区二期启动建设，农副产品国际物流中心及北汽集团配套仓储中心项目正在招商，收费站西迁工程完成。飞虹大桥及飞虹大道等完成项目前期工作。洋湖水厂搬迁、陶瓷博物馆改扩建等工程开工。争取国家和省专项资金突破10亿元，景德镇直升机列入国家战略性新兴产业区域集聚发展试点，当年获得中央资金1.6亿元。发电厂2台100万千瓦机组扩建项目列入我省电力中长期发展规划。

　　（五）城市面貌明显改善。以优化布局、拓展功能、提升品位为目标，全力实施城市建设攻坚战。中心城昌南拓展区以高强度高速度之势全面铺开。南片区路网15条道路27.8公里路基贯通，国内领先、全省第一的地下综合管沟基本建成，西山路、瓷都大道排水系统改造基本完成，总投资200亿元的绿地国际陶瓷文化旅游城启动建设，昌南实验学校、新成大厦等公建项目开工。老城保护与利用工程有序推进。御窑厂国家考古遗址公园初具规模，彭家上弄老街区改造、明清窑作群保护开发、落马桥元青花窑址考古发掘等工作初见成效，陶溪川国际陶瓷文化产业园核心区即将完工，体现瓷都特色和历史风貌的珠山中路综合改造基本完工。高铁商务区和西瓜洲片区建设规划编制基本完成，其它前期工作抓紧进行。着力完善市政设施。瓷都大桥维修、迎宾大道二期改造等一批主次干道及排水系统改造基本完成，西城区截污干管和备用水源工程即将开工。深入开展“五城同创”。全面整治市容市貌、规范陶瓷市场、治理交通拥堵、持续打击“两违”、提升市民素质，取得明显成效，顺利通过全国文明城市提名资格测评。

　　（六）现代服务业加快发展。实现服务业增加值254亿元，增长7%。文化遗产、近现代工业遗存保护和开发力度加大，申遗工作取得重大突破，荣膺“世界手工艺与民间艺术之都”，成为联合国教科文组织全球创意城市网络正式会员。接待国内外游客突破2500万人次，旅游总收入突破200亿元，A级景区从11个增加到18个，乐平怪石林升格为4A景区，瑶里镇入选“中国十大最美乡愁旅游目的地小镇”，丽阳镇入选“古色江西—江西十大文化古镇”。民航客、货运吞吐量分别增长14.5%和186.6%，创历史新高。金融机构存款744.25亿元，增长11.1%，居民人均储蓄存款全省第三；贷款余额423.8亿元，增长17.5%。“财园信贷通”、“惠农信贷通”、“出口信贷通”累计发放贷款5.8亿元。电子商务发展迅猛，网店总量突破1万家，成交额46.8亿元，增长1.25倍。

　　（七）各项改革不断深化。行政区划平稳调整到位，基本形成界限清晰、布局合理、发展共赢的格局。机构改革全面启动实施。精简行政审批事项259项，精简率60.7%，出台了市本级行政审批权力清单；网上审批试点取得成效，建设项目并联审批有序推进，网上“中介超市”上线运行。数字化综合办公平台基本建成。国资国企监管体制改革取得成效。财税体制改革深入推进，国库集中支付基本实现全覆盖，财政预算绩效管理全面实施，政府向社会购买服务试点不断扩大。公务用车制度改革、事业单位分类改革等完成调查摸底。农村集体建设用地和宅基地调查确权进展顺利，农村土地承包经营权确权登记扎实推进，农村集体经济组织产权制度改革试点全面启动。国有林场改革基本完成。

　　（八）民生及社会事业协调发展。在财政压力持续加大的情况下，安排民生资金84.6亿元，占公共财政预算支出56.6%，为民办10件实事基本完成。城镇就业率96.5%，城镇新增就业3.98万人；新增转移农村劳动力2.96万人。养老、医疗、工伤、生育保险征缴收入创新高。加快棚户区和农村危房改造，保障房建设基本实现“三年任务一年完成”的目标。基本普及学前三年教育，职教就业率连续三年超过95.2%，新建、改造了一批校舍和设施。成功承办中国文化遗产日主场城市活动；南窑遗址考古发掘被评为全国十大考古新发现；寿安金家坞龙窑和湘湖进坑瓷石矿洞遗址的发掘，填补和完善了我市宋代制瓷业体系。景德镇中外陶瓷艺术家协会正式成立。 602所荣获一项国家科技进步一等奖，乐平工业园成为国家精细化工高新技术产业化基地。医药卫生体制改革取得新成效，重大疾病防控进一步加强。十四届省运会金牌总数第三、团体总分第四，争取到第十五届省运会举办权。创建“全国残疾人文化体育示范市”通过国家验收。组织开展了“利剑行动”、“三打两治”等专项行动，“平安瓷都”建设成效显著，拦门堵路、医闹等不法或不当行为有效遏制。乐平市被评为全国文化先进县，珠山区被评为全国和谐社区建设先进城区。进京、赴省、赴市访“三下降”。连续13年未发生重大及以上安全生产事故，非法小煤窑连续8年“零开采、零死亡”。积极支持部队建设和军事斗争准备，加强民兵预备役建设，军转干部、退役士兵及随军家属妥善安置。同时，审计、统计调查、民族宗教、外事侨务、市场监管、供销、老龄、人防、档案、地方志等各项工作也取得了新的进步。

　　一年来，我们坚持以开展党的群众路线教育实践活动为契机，严格执行“八项规定”，全市“三公”经费支出下降31.6%，清理“吃空饷”人员468人；坚持依法行政，完善科学民主决策制度，行政行为进一步规范；加快转变职能，切实改进作风，政府自身建设不断加强。

　　各位代表，以上成绩的取得，是全市人民共同努力的结果。在此，我代表市人民政府，感谢全市人民的信任和支持！感谢人大依法监督和政协民主监督！感谢市内外各界人士对景德镇发展的关心和参与！并向你们表示崇高的敬意！

　　回顾过去的一年，我们也看到，在发展中还有不少困难和问题，主要表现为经济总量较小、财力薄弱、负债较重、要素制约瓶颈仍然存在。从根源上分析，还是缺少一批支撑发展、引领未来的支柱产业和龙头企业，支柱产业在工业经济中的“挑大梁”作用还不明显，传统产业比重较高，创新能力不强，转型升级任务较重。此外，城市建设管理任务艰巨，社会治理能力和水平亟待提高，改善民生任务比较艰巨，政务环境有待进一步优化。对此，我们将高度重视，努力克服和解决。

　　二、2015年工作安排

　　今年政府工作的总体要求是：全面贯彻党的十八大和十八届三中、四中全会以及习近平总书记系列重要讲话精神，深入落实省委十三届十次全会和全省经济工作会议精神，持之以恒贯彻省委十六字方针，准确把握经济发展新常态，增强加快发展的定力，持续深入实施好“三大战役”质量提升年活动，坚持好为标准、快字当先、量质双升，狠抓改革攻坚，突出创新驱动，加强民生保障，努力实现经济健康较快发展和社会和谐稳定。

　　今年经济社会发展的主要预期目标是：生产总值增长8.5%，财政总收入增长9.5%，规上工业增加值增长9.5%，固定资产投资增长15%，社会消费品零售总额增长10%，实际利用外资增长10%，外贸出口增长3%，城镇居民人均可支配收入增长10%，农村居民人均可支配收入增长11%，居民消费价格涨幅低于3%，单位生产总值能耗下降3.5%、二氧化碳排放完成省政府下达目标，人口自然增长率低于8.3‰。

　　重点做好七个方面工作：

　　（一）坚持工业强市战略，培育特色支柱产业

　　全力主攻主导产业。以骨干企业为龙头，延伸产业链，壮大航空、汽车、陶瓷三大主导产业，加速产业集聚。1、推进直升机产业集群建设，加快直升机产业推介会签约项目的落地投产，倾力支持昌飞、602所和江直投、昌兴航空等企业发展，努力打造较为完备的直升机产业链和技术创新体系，吕蒙总装园基本完成建设任务。2、全面深化与北汽集团的战略合作，加速新昌河汽车总部基地建设，全产业链发展汽车产业，新基地实现新车下线，老厂区力争总产突破15万辆。3、通过文化内涵、科技手段、经营业态、营销方式、资本运作等创新，推动陶瓷产业转型升级，壮大日用瓷骨干企业，加快发展高档手工制瓷，培育高技术陶瓷，继续保持艺术陶瓷领先地位。建成红叶陶瓷项目并力争投产，开工建设邑山陶瓷工业综合体，名坊园正式开园。4、稳步推进制冷压缩机及冰箱产业升级，提高精细化工及生物医药产业的资源高效循环利用水平。

　　提升园区承载能力。加快推动市域经济一体化，努力将园区打造成为工业经济的增长极、招商引资的主平台。1、坚持产城融合。加快完善路网等公共基础设施，着手启动商务区、生活区等功能区建设，不断完善园区生产生活功能。2、坚持集约发展。提高土地使用效益，根据用途最佳、效率最高、效益最大的原则统筹安排工业用地，并加强低效闲置土地清理，促进集中、集约、节约发展。3、坚持合作共赢。打破各自为战的格局，破除体制障碍，研究出台县区与园区之间“平台共建、利益共享、合作共赢”的管理办法。4、坚持绿色发展。严格环保准入，加大减排力度，强化日常监管，严厉打击环境违法行为，推动发展方式向绿色循环低碳转变。

　　提高工业服务水平。坚持精力向工业聚焦、要素向工业集聚，持之以恒培育骨干企业。1、完善帮扶机制。在健全市领导挂点帮扶机制的基础上，建立每月协调、每季调度制度，掌握重点行业、重点企业、重点产品的生产经营状况，及时帮助企业解决困难。2、倾力扶优扶强。对焦化、华意等生产规模大、创税能力强的龙头骨干企业，实行“一企一策”，优先保障用地、融资及水电气运；同时优化施工环境，严厉打击非法阻工和强揽工程、强买强卖等违法行为。3、强化创新驱动。突出企业技术创新主体地位，推进产学研协同创新，并通过管理和商业模式创新，推动传统产业转型升级。

　　（二）全方位扩大开放，提高项目建设水平

　　提升招商引资成效。1、强化产业集群招商。重点围绕航空、汽车、陶瓷、医药化工等优势产业，积极引进航空制造、汽车零部件等配套企业，完善产业链条，打造功能集聚、竞争力强的产业集群。同时，开展物流、电子商务、旅游、文化创意等现代服务业以及现代农业招商。2、集中精力招大引强。梳理工业产业、现代农业、新型业态等重点产业链上的薄弱环节，对接国内外 500强、央企以及高成长性企业，积极引进龙头型、税源型、产业带动力强的大项目好项目。3、改进招商方式。在完善小分队招商机制的同时，组建主导产业专业招商队；通过政府驻外机构、在苏浙闽等地建立招商联络处及驻外商会等渠道，大力开展驻点招商。4、扩大外贸出口。融入“一带一路”战略，鼓励企业开拓国际市场，重点扶持生产型企业扩大陶瓷、茶叶等产品出口。

　　积极争取上级支持。在科学编制“十三五”规划的同时，加强宏观形势和政策研究，精心策划、包装项目，更多地争取上级支持。力争直升机产业、昌景黄高速铁路、机场搬迁等重大项目列入国家或省“十三五”规划；争取第三轮转型政策延续，并加大转移支付力度、延长转移支付年限；争取将邑山陶瓷工业综合体项目列入全国城区老工业区整体搬迁改造范围。向省积极争取直升机产业创业投资基金。

　　强化基础保障能力。加快浯溪口大坝主体工程建设，确保汛前具备挡水条件、第一台机组具备安装条件。按照时间节点推进九景衢铁路建设。防洪工程吕蒙堤段完成主体工程建设任务、瓷都大桥至观音阁段完成堤后城市道路工程。完成206国道大中修。加快洋湖水厂搬迁、樟树坑引水工程建设。开工建设飞虹大桥及飞虹大道。跟踪推进发电厂2台100万千瓦机组扩建项目。力争皖赣铁路扩能项目开工，并抓紧昌景黄高速铁路、六安景铁路项目前期工作。

　　（三）同步推进扩容提质，着力提升城市形象

　　高质量建设中心城区。1、全速推进中心城昌南拓展区建设。基本建成南片区路网并投入使用，全面启动北片区路网和西河防洪堤建设，基本建成学校、社会养老服务中心、新成大厦等公建项目，开工建设绿地瓷展会馆、博能艺术城等产业项目。2、有序实施老城区保护和利用工程。编制老城区“三旧”改造提升五年规划。完成老街区房屋修缮、落马桥“三红一光”项目规划设计等前期工作，修复一批古窑业遗址，建设明清窑作群陶瓷文化遗存展示馆，陶溪川项目核心区建成开园。3、加快建设赣东北综合物流园。完善园区洪源一路、长安大道往南延伸等路网，加快向南扩园，开工建设集长途客运、公交、旅游集散等功能为一体的综合交通枢纽项目，并加大物流企业招商力度，尽快形成规模。4、全面推开高铁商务区核心区建设。开工建设站前广场、高铁大道、站前一路等项目。同时，陆续启动景东、韭菜园、西瓜洲片区建设。

　　加快完善城市功能。开通月亮湖景南高速出口，拓宽206国道月亮湖至昌南大道段、景德大道豪德至白鹭大桥段，创造条件争取启动景德大道延伸至湘湖段工程，完成景东大道二期建设，形成城市外围快速路网，并打通一批城市断头路。完成中心城区部分主次干道人行道改造。继续实施已立项的城区排水改造工程，完成老南河疏通改造。对人民广场、昌江广场进行改造。实施省级（陶瓷创意）商贸示范街改造工程。编制城区排水专项规划，完成第二污水处理厂截污管网、宋家山垃圾处理厂扩容等工程。争取垃圾焚烧发电厂早日运行。

　　提升城市管理水平。1、强化规划引领作用。编制完成历史文化名城规划并加快实施，抓紧编制老城区保护利用规划，启动电力、通讯、地下空间等专项规划编制工作，实现市政基础设施和公共服务设施专项规划全覆盖。同时，积极开展“多规合一”试点。2、着力解决“脏乱堵”问题。严格落实“门前三包”责任制，开展环境卫生、户外广告、占道经营、渣土运输、陶瓷市场、机动车停放等专项整治。继续严厉打击“两违”，完善控违长效机制。加强“青山白化”整治，引导市民文明丧葬，革除乱埋乱葬陋习。3、深入开展“五城同创”。以创建全国文明城市为统揽推进创建工作，力争完成国家卫生城市申报，启动创建国家森林城市八大工程，重点打造以珠山中路为核心的文明创建示范区。建立健全志愿者服务和城市管理监督员等长效机制。4、规范房地产和建筑市场秩序。开展规划专项整顿，完成房地产市场清查和直管公房清理整顿。

　　（四）加快农业现代化，促进县域经济升级

　　提高农业综合生产能力。完成规划内92座病险水库除险加固，实施新出险37座小型病险水库除险加固，加快实施8座中小河流治理乡镇防洪工程。切实做好基本农田划定工作，加强耕地保护。继续推进农业三大体系建设，整合资金建设高标准农田9.2万亩，粮食播种面积和总产量分别稳定在139万亩和12亿公斤以上，完成造林4.2万亩。

　　积极发展现代农业。引导现代生产要素向农业农村流动，引导龙头企业向蔬菜、茶叶等优势产业集聚，积极培育新型农业经营主体，发展多种形式的规模经营。加快乐平市国家级现代农业示范园建设。新增农业产业化市级以上龙头企业10家，市级以上龙头企业实现销售收入55亿元。争创国家级休闲农业示范点1个，省级休闲农业示范点2个。建设标准化菜园基地、蔬菜集约化育苗场，实现蔬菜播面49.5万亩、产量115万吨。壮大提升浮梁茶产业，作响茶叶品牌，实现茶园面积15万亩、产量6500吨。

　　建设和谐秀美乡村。把镇村联动作为城乡一体化的关键环节，重点抓好中心镇、特色镇、中心村、特色村、城中村建设，继续抓好3条美丽乡村综合示范带联动建设。新农村建设重点由基础村转向中心村，着力提升新农村建设点品质。加快浯溪口水库移民搬迁，妥善安置移民。开展农村人居环境整治，重点实施农村清洁工程，逐步实现垃圾处理城乡一体化。浮梁县要按照国家生态县的建设要求，实现经济社会和生态环境协调发展。

　　发展特色县域经济。乐平市重点打造精细化工和医药、机械电子、蔬菜及食品加工产业链，走经济效益高、环境污染少的高效生态产业发展之路。浮梁县要创新绿色发展模式，在精心打造茶叶和旅游品牌的同时，紧紧依托昌河汽车新基地，围绕延伸汽车产业链发展机械电子等配套产业。昌江区要主动承接城市辐射，加快发展商贸物流、休闲旅游、特色农业，在深化与高新区的合作中壮大工业实力。珠山区重点发展商贸服务、楼宇总部经济两大支柱产业，并大力发展文化旅游、电子商务，促进城市经济繁荣。

　　（五）拓展现代服务业，优化产业发展结构

　　推进旅游强市建设。围绕打造国际旅游名城的目标，促进旅游与陶瓷、文化、商贸等产业融合，发展旅游产业集群，力争旅游总收入240亿元，增长20%。1、加强旅游景区创建。按照“城市即景区、景区即城市”的思路，打造城市旅游新业态。支持高岭·瑶里创建5A级景区，将陶瓷工业园、陶溪川、三宝瓷谷等建成陶瓷文化旅游新景区，创建一批陶瓷文化A级景区。推进严台、沧溪等历史文化名村建设。2、扩大旅游营销。利用赣东北旅游区域联盟、闽浙赣皖九方经济区等平台，加强旅游区域合作。加快智慧旅游建设，打造数字综合服务平台，充分运用新媒体开展宣传推介。3、改革旅游投融资体制。积极整合旅游资源，着手组建旅游集团。

　　加快发展金融服务业。积极引进招商银行设立分支机构，全力支持市商业银行推进重组。鼓励民间资本参与组建村镇银行、贷款公司、融资担保等新型金融机构，着力发展普惠金融。健全政银企对接机制，鼓励银行业加大对中小微企业和“三农”信贷投放。推进资本市场发展，全力支持世龙实业、富祥药业上市。加快宏柏化工、开门子肥业、昌兴航空、红叶陶瓷改制上市步伐，力争至少1家企业在主板首发上市。鼓励海能燃气、国信节能、江隆汽车消声器等企业在“新三板”挂牌。

　　积极发展其他现代服务业。以获评世界“手工艺与民间艺术之都”为契机，充分挖掘丰厚的文化资源，促进文化产业转型升级。以陶溪川、名坊园、建国创意园等为重点，加快文化创意产业集聚区建设，壮大产业规模、提升产业层次。深化文化领域市场化改革，完善市演艺公司法人治理结构和公司制改造，加快御窑管理处等经营性文化单位市场化步伐。加快发展现代物流、工业设计、技术研发、融资租赁、信息服务等生产性服务业，助推产业向价值链高端提升。积极引进实力较强的专业性会展企业，提升瓷博会办展效益和水平。依托重点产业和优势产品，普及深化电子商务，力争打造一家规模较大的陶瓷电子商务企业。鼓励民营经济发展，引导社会资本投向商贸、教育、娱乐、保健、家政等生活性服务业，满足群众多样化消费需求。积极应对人口老龄化，加快编制养老服务事业发展规划，加强养老服务设施建设，完善养老服务体系。

　　（六）加快各项改革步伐，不断释放市场活力

　　按照全省的统一部署，加快改革步伐，为发展升级注入强大动能。重点推进下列改革：1、完成政府机构改革，确保无缝对接、平稳过渡。2、进一步深化行政审批制度改革，大力推行“三单一网”，启动权责总清单编制工作。3、启动社会信用体系建设，搭建覆盖事业单位、社会组织、企业、个人的信用信息数据库，建立守信激励和失信惩戒机制。4、完善数字化综合办公平台，加快推进无纸化办公，上半年市本级全面投入使用。5、进一步深化国企国资改革，加快兼并重组步伐，积极发展混合所有制经济。完成市公交公司改革重组，启动红叶陶瓷、陶瓷文化创意产业、物流、房地产、建工、商贸等集团公司的组建；加大机关事业单位国有资产统一经营管理力度，增强市国资公司融资能力。6、整合资源培育壮大投资运营公司，组建完成国有资产经营投资控股公司、园区建设开发投资公司、农业产业投资运营公司，壮大城建开发投资公司。7、深化城市管理体制改革。坚持责权利相统一，落实属地管理责任，推进市政、园林管理政企分离，下放环卫等职能，完成区级环卫市场化改革，在市政、园林领域逐步实行服务外包。8、加快陶瓷科研体制改革。整合陶瓷科研资源，创造条件争取兴建“产学研用”实验中心，促进科研成果转化。9、全面启动警务机制改革，力争取得阶段性成果。10、根据全省统一部署，全面完成第一批行政及参公单位公务用车制度改革。11、全面完成农村土地承包经营权确权登记颁证工作，全面开展农村宅基地登记工作。12、完成农村集体经济组织产权制度改革和林地流转改革试点。

　　（七）切实保障民生，统筹社会事业发展

　　着力改善民生状况。加大民生资金投入，实施民生工程。重点为民办好10件实事：1、为困难群众购买公益性就业岗位2100个，新增发放小额贷款3.85亿元；2、新农合省、市级定点医疗机构住院补偿比例提高到55%，启动城乡居民健康一卡通工程；3、开工建设1座老城区跨江大桥；4、规划建设4个停车场，实施10条老城区道路建设和改造；5、购置100辆新能源空调公交车，新建（改造）100座公交候车亭；6、开工建设1995套公租房，改造棚户区5428套（户）；7、新建官庄农贸市场，改造黄泥头、中华北路等3个农贸市场； 8、新建5个城市公园（街心花园）； 9、建成8座站厕合一垃圾中转站，改造15座中心城区旱厕；10、实施卫星直播户户通工程，让7.2万户边远群众接收到广播电视。

　　协调发展社会事业。强化企业技术创新决策、投入、成果转化的主体地位，提高科技协同创新水平；加强科技创新平台建设，促进科技与金融结合；申报国家级产学研产业联盟试点，支持乐平工业园区申报省级高新技术开发区。深化教育领域综合改革，优化城乡中小学布局，加快义务教育学校标准化建设，推进优质教育资源扩充工程，积极构建现代职业教育体系。深化公立医院改革，提高重大疾病防控能力，整顿医疗市场秩序。整合食品安全监管资源，加强食品、药品、餐饮卫生监管，保障群众健康安全。全面落实与故宫博物院的合作协议，积极开展各项合作活动。加强馆藏文物管理。积极备战十五届省运会，加快场馆设施建设改造步伐。加快实施“数字化档案馆”项目。把国防动员工作摆在突出位置，促进军民融合深度发展，继续做好民兵预备役等工作，争创“全国双拥模范城”六连冠。

　　提高社会治理水平。深入推进“法治景德镇”建设，推动社会治理从政府单向管理向政府主导、社会多元主体共同治理转变。完善立体化社会治安防控体系，夯实防控基层基础。运用新一代信息技术，整合政府管理和公共服务资源，加快建设“智慧城市”，有效提升政府管理能力，促进市民生活更加智能便捷。完善网上信访、视频接访系统，全面推进市直部门矛盾纠纷调处中心和基层“百姓说事中心”建设，有效预防和化解社会矛盾。加大网络监管力度，及时引导网络舆论，凝聚网络正能量。深化安全生产“打非治违”和重点行业领域专项整治，增强安全生产监管监察、技术支撑和事故应急处置能力，防止发生重特大安全生产事故。

　　各位代表，今年是全面推进依法治国的开局之年，“十二五”收官和“十三五”奠基的承前启后之年，也是我市“三大战役”的质量提升之年。新的形势和任务，对政府自身建设提出了更高要求。为此，我们将进一步解放思想。以自我革新的勇气和胸怀，冲破旧思想旧观念的障碍，跳出条条框框限制，克服部门利益掣肘，以思想的大解放，促进政务环境大改善，推动经济社会大发展。我们将提高政府法治化水平。按照建设法治政府的要求，坚持用法治思维和法治方法履行政府职能，依法规范行政行为，推动改革发展。我们将加快转变政府职能。继续大力简政放权，提高工作效率和服务质量，拓展服务内涵，营造公平竞争的市场环境，充分激发市场活力和社会创造力。我们将持续改进工作作风。强化大局观念、担当意识、进取精神，切实增强执行力、操作力和破解难题的能力，确保政令畅通、决策落地生根。我们将强化廉政建设。认真落实“一岗双责”，坚持用制度管权管人管事，以“零容忍”态度坚决查处违法违纪行为，努力打造高效政府、务实政府、廉洁政府。

　　各位代表，做好今年的工作，意义十分重大。让我们在省委、省政府和市委的坚强领导下，紧紧依靠全市人民，坚定信心，保持定力，持续发力，久久为功，为实现“五年决战同步小康”奠定坚实的基础，作出更大的贡献！
